# Joint modelling of serological and hospitalization data reveals that high levels of pre-existing immunity and school holidays shaped the influenza A pandemic of 2009 in The Netherlands

**DOI:** 10.1098/rsif.2014.1244

**Published:** 2015-02-06

**Authors:** Dennis E. te Beest, Paul J Birrell, Jacco Wallinga, Daniela De Angelis, Michiel van Boven

**Affiliations:** 1 Centre for Infectious Disease Control, National Institute for Public Health and the Environment, PO Box 1, Bilthoven 3720AB, The Netherlands; 2 MRC Biostatistics Unit, Cambridge Institute of Public Health, CB2 0SR

**Keywords:** Bayesian evidence synthesis, influenza A, serology, hospitalization incidence, mixture analysis, transmission model

## Abstract

Obtaining a quantitative understanding of the transmission dynamics of influenza A is important for predicting healthcare demand and assessing the likely impact of intervention measures. The pandemic of 2009 provides an ideal platform for developing integrative analyses as it has been studied intensively, and a wealth of data sources is available. Here, we analyse two complementary datasets in a disease transmission framework: cross-sectional serological surveys providing data on infection attack rates, and hospitalization data that convey information on the timing and duration of the pandemic. We estimate key epidemic determinants such as infection and hospitalization rates, and the impact of a school holiday. In contrast to previous approaches, our novel modelling of serological data with mixture distributions provides a probabilistic classification of individual samples (susceptible, immune and infected), propagating classification uncertainties to the transmission model and enabling serological classifications to be informed by hospitalization data. The analyses show that high levels of immunity among persons 20 years and older provide a consistent explanation of the skewed attack rates observed during the pandemic and yield precise estimates of the probability of hospitalization per infection (1–4 years: 0.00096 (95%CrI: 0.00078–0.0012); 5–19 years: 0.00036 (0.00031–0.0044); 20–64 years: 0.0015 (0.00091–0.0020); 65+ years: 0.0084 (0.0028–0.016)). The analyses suggest that in The Netherlands, the school holiday period reduced the number of infectious contacts between 5- and 9-year-old children substantially (estimated reduction: 54%; 95%CrI: 29–82%), thereby delaying the unfolding of the pandemic in The Netherlands by approximately a week.

## 1. Introduction

Worldwide, influenza A causes considerable morbidity and mortality in years with high influenza activity [1,2]. Proper assessment of the epidemiological dynamics is key for effective control. However, it is not uncommon that different data sources yield conflicting information [3]. For instance, influenza-like illness surveillance through networks of general practitioners showed increases in incidence in many countries well before increases in seropositivity and virus isolation rates, possibly because of increased public awareness. The advent of modern statistical methods combined with explosive increases in computing power has enabled systematic integration of different data sources in unifying statistical frameworks, thereby providing proper weighting of the various types of data.

In this paper, we analyse the transmission dynamics of the influenza pandemic of 2009 by linking a dynamic transmission model to serological and hospitalization data in a Bayesian inferential framework. The serological data provide information on levels of immunity and infection attack rates, and the hospitalization data give an indication of the timing and severity of the epidemic in different age groups. The model classifies persons in one of four stages of infection (susceptible, exposed, infectious and recovered) and describes the transmission dynamics of influenza A in an age-structured population [3–5]. In the model, contacts between persons of different age are made at rates that are determined by observed human contact patterns [6].

The serological data consists of two cross-sectional surveys involving random samples from the Dutch population, one performed just before and the other just after the pandemic [7,8]. As in an earlier study, we model these data with mixture models in which each sample has a certain probability of belonging to a person who was susceptible, previously exposed and immune, or recently infected [7]. This has a distinct advantage over the more rigid classification of persons as being either susceptible, immune or infected as it takes into account biological variation in antibody concentrations. In the mixture modelling approach, there is no need to set a specific threshold for classification, even though optimal thresholds can be derived from the data [9]. Consequently, not only do the serological data inform the hospitalization data, but the hospitalization data also inform classification of serological samples. In other words, probabilistic classification of serological samples is determined by the information contained in both the serological and hospitalization data.

Based on the high contact rates of children, we expect an epidemic to peak first in children [5,6,10]. An unusual pattern observed in the Dutch hospitalization data for the 2009 pandemic is that the epidemic peak in young children occurred relatively late. This may have been caused by the one-week holiday in weeks 43–44, a couple of weeks before the pandemic peak. It is known that school holidays can considerably reduce the number of contacts made by children, thereby reducing transmission [11,12]. We use the inferential framework sketched above to estimate age-specific infection and hospitalization rates and to investigate to what extent the delayed peak in young children can be explained by school holidays.

## 2. Material and methods

### 2.1. Serological data

**Figure 1.**
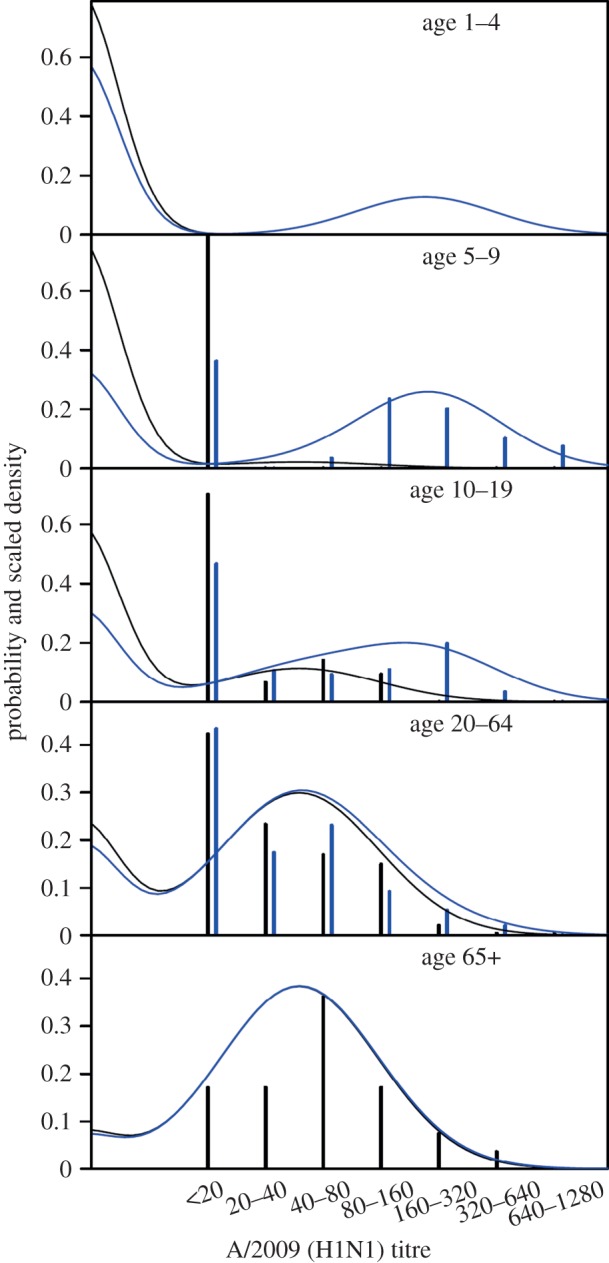
Overview of the serological data (bars) and fit of the model with school holiday (lines). Panels show results for the various age groups. Bars show the serological data aggregated in titre classes (<20, 20–40, 40–80, 80–160, 160–320 and 320–640). Black bars and black lines denote pre-pandemic data and pre-pandemic model fit, respectively. Blue bars and lines show the post-pandemic data and post-pandemic model fit. Note that no serological data are available in young children (1–4 years) and that only pre-pandemic data are included in the oldest age group (65+ years).

In The Netherlands, two cross-sectional serological surveys were conducted before and after the pandemic of 2009 [7]. In this study, samples were tested by a haemagglutination inhibition test (HI) to estimate age-specific infection attack rates. A structured random subset of the samples was subsequently tested against a panel of antigens using an antibody protein micro array (figure 1) [8]. The protein micro array is a novel diagnostic assay to investigate antibody responses to subunit 1 of the haemagglutinin surface glycoprotein (HA1) [13–16]. Analyses show that the micro array is more sensitive and more specific than HI in distinguishing recent infection from prior exposure [8,13]. Infection attack rates estimated with the micro array are similar to those estimated with HI, but are more precise for a given sample size [8].

A total of 357 people were tested with the micro array; 167 in the pre-pandemic study and 190 in the post-pandemic study. The age of the participants ranged from 5 to 75 years. Data were stratified according to the following age categories: 5–9, 10–19, 20–64 and 65+ years, following recommendations of the Consortium for the Standardization of Influenza Seroepidemiology (consise.tghn.org), with the exception that the age groups 20–44 and 45–64 years were aggregated. The samples in the pre-pandemic subset were collected before 12 October 2009, i.e. well before the onset of widespread influenza circulation [7,8,17]. The serological data are available in the electronic supplementary material.

The post-pandemic survey was carried out several months after the pandemic period; and from these data, we excluded people who were vaccinated against the pandemic virus [7]. Exclusion of persons from the post-pandemic survey who had a pandemic vaccination also led to the exclusion of many persons with a history of seasonal vaccinations [8]. Due the underrepresentation of persons with a history of seasonal vaccination in the post-pandemic sample in the oldest age group (65+ years), this subset of the data does not represent a random sample from the population and was not included in the analyses [8]. As a result, serological data in the oldest age group cannot directly be used to estimate the attack rate. However, the pre-pandemic sample of the oldest age group does represent a random sample of the population and was used in the statistical analyses.

### 2.2. Hospitalization data

During the pandemic, all hospitals in The Netherlands were required to notify the municipal health services of hospital admissions of patients with a laboratory-confirmed influenza A infection. Our hospitalization data represent the total, nationwide daily number of hospitalizations, i.e. the daily number summed over all municipal health services [17]. We use the same age stratification for the hospitalization data as for the serological data (5–9, 10–19, 20–64 and 65+ years), supplemented with children aged 1–4 years. Upon admittance to a hospital, persons were asked when their symptoms had started. The number of hospitalized influenza cases by day of symptom onset provides direct information on the epidemic curve. A total of 1610 cases was available for analysis, distributed over the age categories as follows: 1–4 years: 267 cases; 5–9 years: 196 cases; 10–19 years: 225 cases; 20–64 years: 800 cases; and 65+ years: 122 cases. The average time between onset of symptoms and admittance to the hospital was 2.4 days.

### 2.3. Transmission model

The hospitalization and serological data are linked through a Susceptible–Exposed–Infectious–Removed transmission model [5], thereby providing a natural weighting of the different types of data. In the model, the exposed and infectious periods are modelled using Erlang (gamma) distributions, yielding control over variation in the exposed and infectious stages [5,18]. Specifically, we include four exposed and four infectious stages, so that distributions of time in the exposed and infectious classes have means 1/*η* and 1/*γ*, and shape parameters *n*_E_ = *n*_I_ = 4, respectively.

In each of the five age groups, individuals make contact with individuals in other age groups at rates that are proportional to a mixing matrix ***C***, which is specified by observed human contact patterns [6]. As in earlier analyses, a proportionality parameter *z* reflects the probability of transmission per contact. Hence, the basic reproduction number in a susceptible population and the reproduction number in a population with pre-existing immunity are given by the spectral radiuses of *z**C*** and *z**CS***, respectively, where age-specific proportions that are initially susceptible are collected in the matrix ***S*** [19].

Further, if we denote by *R_a_* the relative frequencies of removed individuals in age group *a*, and by *E_a,j_* and *I_a,j_* the relative frequencies of exposed and infectious individuals in age group *a* of stage *j* (see below), then the model dynamics is specified by the following differential equations:2.1
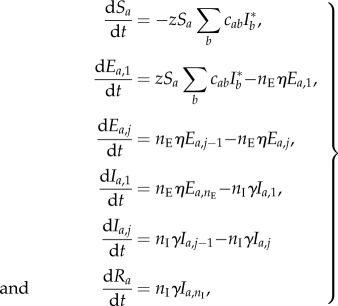
where the index *a* runs through all age groups, the index *j* runs through all exposed and infectious stages except for the first (*j* = 2, … ,*n*_E_ for dE_*i*,*j*_/d*t*, and *j* = 2, … , *n*_I_ for dI_*i*,*j*_/d*t*) and 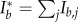 is the total relative frequency of infectious individuals in age group *b*. In line with empirical evidence, the average lengths of the exposed and infectious periods are both set to 1.5 days [20]. In this manner, the generation time is 1/*η* + 1/*γ* (*n*_I_ + 1)/2*n*_I_ = 2.4 days [21].

The fractions of immune persons at the start and end of the pandemic are given by *R_a_*(0) and *R_a_*(∞), and these quantities are estimated in age groups with serological data (5–9, 10–19, 20–64 and 65+ years). No serological data are available in the youngest age group (1–4 years). It is, however, plausible that there was very little pre-existing immunity in this age group, so we take *R*_1–4_(0) = 0. The fractions of immune persons at the start and end of the pandemic feed into the mixture model (see below).

### 2.4. Hospitalizations likelihood

The hospitalization incidence data provide the timing of symptoms onset for confirmed influenza A infections requiring hospitalization. Making the reasonable assumption that the onset of symptoms is close to the point at which people become infectious [20], we can directly relate the incidence 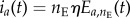 in age group *a* at time *t* to the hospitalization data. Alternative assumptions, e.g. assuming that the onset of symptoms occurred halfway through or at the end of the infectious period (i.e. assuming *i_a_*(*t*) = *n*_E_*ηI_a_*_,2_(*t*) or *i_a_*(*t*) = *n*_E_*ηI_a_*_,4_(*t*)) result in a minor shift of onset of symptoms and yield virtually identical results (not shown). Assuming further that the expected number of hospitalizations is proportional to the numbers of infections, the expected number of hospitalizations at time *t* in age group *a* is given by2.2
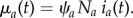
where *N_a_* denotes the number of individuals in age group *a*, and *ψ*_a_ is the age-dependent probability of hospitalization per infection. Thus, the log likelihood of the hospitalization data *H_t,a_* is given by2.3
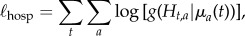
where the indices *t* and *i* run through all observation periods (i.e. weeks 41–51) and age groups (1–4, 5–9, 10–19, 20–64 and 65+ years), and *g*(*H_t_*_,*a*_|*μ_a_*(*t*)) is the probability density function of the hospitalizations. Throughout, we assume that hospitalizations are Poisson distributed.

### 2.5. Serology likelihood

The micro array data are fitted with a Gaussian mixture model that consists of three normally distributed components for the log-transformed serological data. The first and second components correspond to samples with antibodies present in low and intermediate concentrations, representing susceptible individuals and individuals with pre-existing immunity, respectively. The third component corresponds to samples with high antibody concentrations, consistent with recent A/2009 (H1N1) infection. Motivated by biological considerations, we fit the distributions of persons who were susceptible or had pre-existing immunity to the pre-pandemic data, and all three distributions to the post-pandemic data [7,8]. For each distribution, we estimate a mean and standard deviation ***θ****_i_* = (*μ_i_*, *σ_i_*). Densities of the distributions are denoted by *f_i_*(*x*; ***θ****_i_*) or simply *f*(*x*; ***θ****_i_*).

Age-specific mixing parameters are collected in vectors **p** and **q**. Here, *p_a_* represents the fraction of the population in age group *a* that has pre-existing immunity, and *q_a_* is the fraction in age group *a* that has been infected. Hence, 1 −*p_a_* and 1 −*p_a_* −*q_a_* are the corresponding pre- and post-pandemic fractions of susceptible persons in age group *i*. Further, we denote by *g_i_* the age label of sample *i*, by *n*_pre_ and *n*_post_ the number of samples in the pre- and post-pandemic surveys, by *d*_pre,*i*_ and *d*_post,*i*_ the log_2_ antibody titres in the pre- and post-pandemic studies and by *w_i_* the contribution of sample *i* to the population census [7,8]. With these notational conventions, the log likelihood of the pre-pandemic data is given by2.4
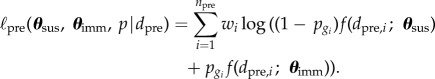
In similar fashion, the log likelihood of the post-pandemic data is given by2.5
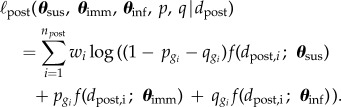
Note that the fraction of persons with pre-existing immunity in the pre-pandemic study equals the fraction of persons with pre-existing immunity in the post-pandemic study. This assumption is reasonable, given that there was only a short-time span between the two studies (less than six months) making significant loss of immunity unlikely. Combining the above, the log likelihood of the serological data is given by *ℓ*_pre_ + *ℓ*_post_, and the total likelihood of the joint hospitalization data and serological data is given by *ℓ*_hosp_ + *ℓ*_pre_ + *ℓ*_post_.

The age-dependent weights of the three mixture distributions have an epidemiological interpretation, representing the fractions of the population that were initially susceptible, had pre-existing immunity and had been infected (equation (2.5)). The weights are linked to the epidemic model via equation (2.1) as follows. In the epidemic model, the fractions of susceptible and immune persons before the epidemic are given by *S_a_*(0) and *R_a_*(0), the number of immune and infected persons at the end of the pandemic is given by *R_a_*(∞), and the attack rate is given by *R_a_*(∞) − *R_a_*(0)*.* In the mixture model, we thus take *p_a_* = *R_a_*(0) and *q_a_* = *R_a_*(∞) − *R_a_*(0) (see the electronic supplementary material for details).

### 2.6. School holiday

In The Netherlands, there is a week-long autumn school holiday, which was planned in week 43 (estimated at 71% of the population) or week 44 (29% of the population). We use those estimates in the analyses with school holiday effect. The 5–9 and 10–19 year-old age groups consist largely of school-going children. The number of contacts within these age groups are discounted during the two weeks of school holiday by replacing the elements *c_ii_* in the contact matrix by *v_t_r_i_c_ii_* + (*i* − *v_t_*)*c_ii_*. Here, *v_t_* represents the fraction of children that have a holiday in week *t*, and *r_i_* represents the contact reduction during the holiday. We estimate separate contact reductions *r_i_* for the two age groups 5–9 and 10–19 years.

### 2.7. Estimation

We considered two main scenarios, one without and the other with the school holiday effect. The models have 16 and 18 parameters to be estimated, respectively (see tables 1 and 2, and the electronic supplementary material, tables S2 and S3). We use a Bayesian framework to enable flexible incorporation of prior information. The parameters specifying the fractions infected and the fractions immune at the start of the epidemic (*R_a_*(0)), specifying the impact of the school holiday (*r_a_*) and determining the fraction of the population at the start of the pandemic are constrained to the domain [0,1], and we use Jeffrey's prior distributions for these parameters. The parameters ***θ***_sus_, ***θ***_imm_ and ***θ***_inf_ are constrained to the domain [0, *∞*], and we assume (improper) uniform prior distributions for these parameters. The hospitalization probabilities (*ψ*_a_) are also constrained to the domain [0, 1]. It is plausible that the *ψ*_a_ should not deviate excessively between adjacent age categories. Therefore, and using information from preliminary analyses, *ψ*_a_ is estimated jointly for the age groups 5–9 and 10–19 years, while *ψ*_1–4_, *ψ*_5–19_ and *ψ*_20–64_ are not allowed to deviate more than a factor of five from each other. For the oldest age group, *ψ*_65+_ is not allowed to deviate more than 10-fold from *ψ*_20–64_. In a sensitivity analysis, we allow these deviations to be doubled to a 10- and 20-fold maximal difference.

**Table 1.** Parameter estimates of the model that does not include the school holiday effect. Parameter estimates are represented by the medians of the posterior distribution.

parameter	age group (years)	estimate	(95% CrI)
fraction immune before the pandemic	1–4	0^a^	
	5–9	0.07	(0.00–0.23)
	10–19	0.25	(0.16–0.37)
	20–64	0.70	(0.61–0.76)
	65+	0.90	(0.76–0.95)
infection attack rate	1–4	0.22	(0.20–0.25)
	5–9	0.48	(0.40–0.52)
	10–19	0.31	(0.25–0.37)
	20–64	0.05	(0.04–0.07)
	65+	0.01	(0.00–0.02)
basic reproduction number		1.9	(1.8–2.3)
reproduction number at the start of the pandemic		1.31	(1.29–1.33)
probability of hospitalization	1–4	0.0012	(0.0010–0.0014)
	5–19	0.00040	(0.00034–0.00048)
	20–64	0.0017	(0.0011–0.0022)
	65+	0.010	(0.0037–0.018)

^a^Pre-pandemic immunity is assumed to be absent in young children (1–4 years).

**Table 2.** Parameter estimates of the model that includes a potential reduction in transmission during the school holiday. Parameter estimates are represented by the medians of the posterior distribution.

parameter	age group (years)	estimate	(95% CrI)
fraction immune before the pandemic	1–4	0^a^	
	5–9	0.08	(0.00–0.24)
	10–19	0.29	(0.20–0.41)
	20–64	0.72	(0.63–0.78)
	65+	0.91	(0.75–0.95)
infection attack rate	1–4	0.28	(0.24–0.33)
	5–9	0.53	(0.43–0.58)
	10–19	0.34	(0.27–0.40)
	20–64	0.05	(0.04–0.09)
	65+	0.01	(0.00–0.02)
reduction of transmission during school holiday	5–9	0.54	(0.29–0.82)
	10–19	0.10	(0.00–0.29)
basic reproduction number		2.2	(2.0–2.6)
reproduction number at the start of the pandemic		1.42	(1.37–1.48)
probability of hospitalization	1–4	0.00096	(0.00078–0.0012)
	5–19	0.00036	(0.00031–0.00044)
	20–64	0.0015	(0.00091–0.0020)
	65+	0.0084	(0.0028–0.016)

^a^Pre-pandemic immunity is assumed to be absent in young children (1–4 years).

Estimates of the parameters are obtained in a Markov chain Monte Carlo framework with a random-walk Metropolis algorithm using Gaussian proposal distributions and with standard deviations set to achieve acceptance ratios in the range 0.20 to 0.35 (see the electronic supplementary material). A 1/10 thinned sample of 200 000 is obtained from the posterior distribution, after a burn-in of 10 000 cycles. Inspection of convergence and mixing is assessed visually. All programs are coded in R 3.1.

## 3. Results

**Figure 2.**
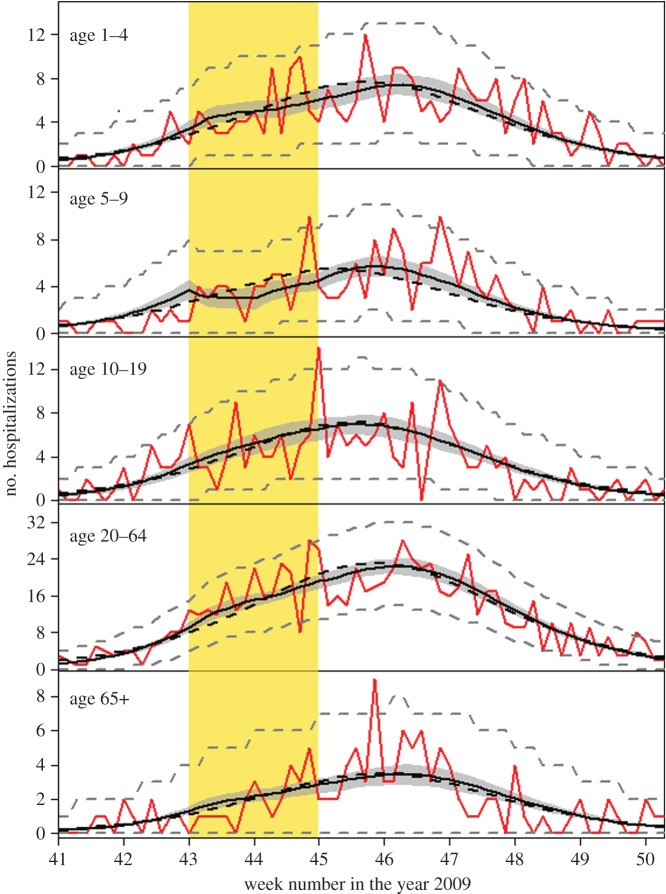
Overview of the hospitalization data (red lines) and model fits (black lines). The red line shows daily incidence of symptoms onset for influenza A requiring hospitalization. The area shaded in yellow indicates the timing of the school holiday. The solid and dashed black lines give fits of the models with and without school holiday, respectively. The shaded black area represents the 95% credible interval of the model with school holiday, and grey dashed lines indicate the Poisson 95% confidence interval of the number of hospitalizations in the model with school holiday.

Figures 1 and 2 give an overview of data and model fits, and parameter estimates of the models with and without school holiday effect are given in tables 1 and 2 and electronic supplementary material, tables S2 and S3. Visual inspection of the hospitalization data and model fits shows that both models adequately describe hospitalizations over time in the different age groups, with no systematic deviations of the predicted from the observed hospitalizations, and no evidence of overdispersion relative to the Poisson model (figure 2). A formal comparison of the two models is made using the Bayes factor (BF), which represents the relative strength of evidence for the model with hospitalization data. To this end, marginal likelihoods are estimated using the harmonic means of the posterior likelihood values of the competing models, yielding stable log-likelihood estimates of −1087.8 and −1096.5. Hence, the comparison indicates that the model with holiday effect is strongly supported by the data [22]. This is due mainly to the fact that the model with holiday effect is able to capture the lagging number of hospitalizations in the ascending phase of the epidemic in 5- to 9-year-old children in weeks 43–44. The predicted contact reduction among children in this age groups is 54% (95% CrI: 29–82%). We do not find a significant reduction among children aged 10–19 years during the school holiday (median reduction 10%, 95% CrI: 0–29%). Incorporating the school holiday not only improved the model fit in children, but also seemed to improve the model fit for the other age groups.

**Figure 3.**
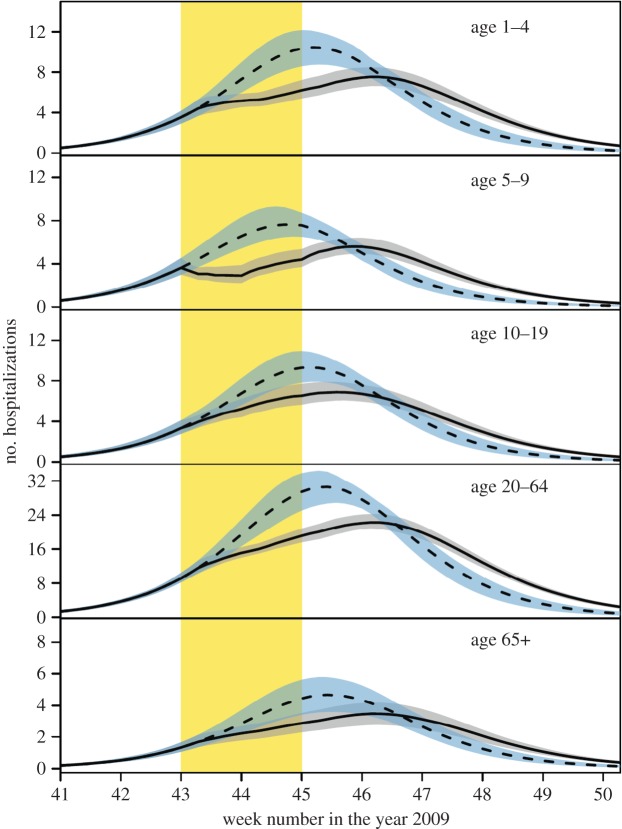
Comparison of model fit (solid black line) with predicted epidemic dynamics in the absence of the school holiday (dashed black line). The area shaded in yellow indicates the timing of the school holiday, and the grey and blue shaded areas represent 95% credible intervals of the model estimate (black) and predicted dynamics without school holiday (blue). The predicted epidemic dynamics was obtained by simulation of the dynamics without school holiday, but with parameter estimates (samples from the posterior distribution) from the model with school holiday (table 2).

To investigate the impact of the autumn school holiday on the epidemic dynamics, we run the transmission model without the school holiday effect with parameters taken from the posterior distribution of the model with school holiday effect. The ensemble dynamics is presented in figure 3. The school holiday appears to have delayed the epidemic peak by approximately one week. Specifically, the estimated delay is 6.9 days (95%CrI: 3.9–9.4) in 1- to 4-year-old children, 7.9 days (95%CrI: 4.9–10.4) in 5- and 9-year-old children, 3.0 days (95%CrI: 0.2–6.2) in 10- and 19-year-old children, 5.4 days (95%CrI: 3.0–7.9) in adults (20–64 years) and 5.5 days (95%CrI: 3.0–7.9) in the elderly (65+ years). Further, our estimates indicate that the holiday period has lowered the epidemic peak by 27% (CrI. 17–35%).

**Figure 4.**
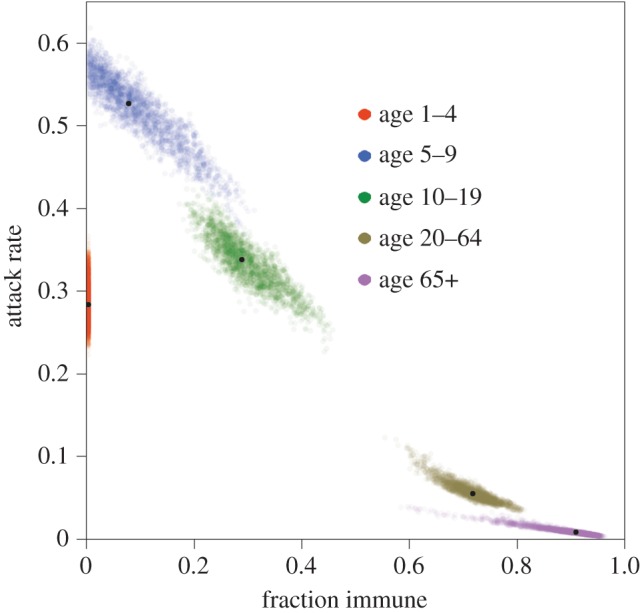
Age-specific estimates of pre-existing immunity and infection attack rates. Coloured dots indicate samples from the posterior distribution, and black dots represent medians of the posterior distribution.

Inspection of the immunity estimates reveal that a large proportion of the adult population had pre-existing immunity and that the high levels of immunity can explain the skewed attack rates (tables 1 and 2). In fact, estimates of the levels of immunity and infection attack rates are strongly correlated (figure 4). Our model also provides estimates of the attack rate in young children (1–4 years), even though no serological data are available for this age group. Specifically, for this age group, the median posterior attack rate is 0.28 (95%CrI: 0.24–0.33).

In the above analyses, the susceptible component of the mixture distribution is located almost entirely below the detection limit (20 U ml^−1^; figure 1), and the immune component has a high density at the detection limit. This implies that samples with an antibody concentration just above the detection limit have a high probability (99% or more) to be classified as immune. We explore how the results are affected if the component of pre-existing immunity is forced to have most (at least 95%) of its weight above the detection limit. The result is an increase in the mean and a decrease in the variance of the immune component (electronic supplementary material, table S4). Estimates of pre-existing immunity in adults remain high, but decrease from 72 to 52% (20–64 years) and from 91 to 84% (65+ years). Likewise, estimates of the infection attack rates remain high in children and very low in adults and the elderly. Estimates of the school holiday effect are negligibly affected.

In a second sensitivity analysis, we allow the probability of hospitalization to differ more between age groups (see Material and methods for details, and the electronic supplementary material, table S5, for full results). The main effect is an increase in the estimated level of pre-existing immunity in the elderly (65+ years), accompanied by a substantial increase in the probability of hospitalization from 0.0084 to 0.017 in this age group. In the younger age groups, the probabilities of hospitalization are marginally affected, indicating that the choice of prior distributions has little impact on the posterior distribution, and that the probability of hospitalization can be identified by the data.

Finally, we analyse how our results compare to a traditional approach in which fixed thresholds determine whether an individual is classified as susceptible, immune or recently infected. In the electronic supplementary material, tables S6 and S7, we used threshold values of 20 and 40 U ml^−1^ for distinguishing susceptible from immune or infected persons. The most conspicuous difference compared with our mixture model analysis is that the estimates of pre-existing immunity are lower and attack rate estimates are higher. Further, estimates of the school holiday effect are consistently higher in the analyses with thresholds than in the mixture model.

## 4. Discussion

Building on earlier work [3], our analyses demonstrate how disease incidence and infection seroprevalence data can be combined in a consistent manner to estimate infection attack rates, levels of pre-existing immunity, the impact of school holidays and probabilities of hospitalization. Our analyses improve on previous approaches by using a mixture model for probabilistic classification of serological samples. We believe that this represents a significant advance over traditional analyses that use predefined fixed thresholds. In fact, there is usually considerable biological variation in antibody response data, leading to classification uncertainties and making analyses using fixed thresholds prone to uncontrollable misclassification (electronic supplementary material, figures S6 and S7). In our mixture model approach, classification uncertainties are propagated to the epidemic model in a natural manner. Consequently, our probabilistic classification of serological samples is determined not only by serological information but also by the disease incidence data. This is the reason why the precision of estimates of the infection attack rates and levels of pre-existing immunity are comparable in the mixture model and the models with fixed thresholds; the lack of classification certainty in the mixture model is compensated by better use of the hospitalization data.

Our results suggest that high levels of immunity in persons older than 20 years, the lack of immunity in persons younger than 10 years and the week-long autumn holiday had a substantial impact on the epidemic in The Netherlands. A weighted average over the estimated contact reduction in 5–9- and 10–19-year-old children (54 and 10%) yielded an estimated contact reduction of 25% in 5- to 19-year-old children, which is similar to the reduction reported by Cauchemez *et al.* [11] for children in France. Our estimate is smaller than the reported reduction of more than 50% in children during the Canadian summer holiday [12]. Possible explanations for the relatively small estimated reduction in 10- to 19-year-old children are that they may be less dependent on school for social contacts and that a small proportion of 10- to 19-year-old children no longer attends school. The difference in the estimates in 5–9- versus 10–19-year-old children is of sufficient magnitude to suggest that the largest reduction in transmission would be achieved by closure of primary rather than secondary schools.

Parameter estimates in our analyses are informed by (i) serological data, (ii) hospitalization data, and (iii) the transmission model armed with age-specific contact patterns. Estimates of the infection attack rates are largely (but not exclusively) informed by the serological data. Particularly for the 5–9- and 10–19-year-old age groups, the post-pandemic serological data are bimodally distributed, and the distribution of infected persons is clearly identifiable (figure 1). On the other hand, attack rates have to be in line with the pandemic model and are restricted in particular by the contact patterns. The result is that confidence intervals of the attack rate estimates are smaller in our analyses than when the attack rates are estimated using the serological data alone (see below). This is especially true for the older age groups in which the serological data *per se* provide little information. Here, the information in the serological data for the younger age groups is carried over to the older age groups via the contact patterns specified by the epidemic model.

In a similar vein, estimates of the levels of pre-pandemic immunity are largely informed by the serological data. In fact, mixture model analyses without hospitalization data are characterized by high estimated attack rates below the age of 20 years (5–9 years: 65%; 10–19 years: 27%) and low attack rates above 20 years (20–44 years: 5% ; 45–64 years: approx. 0%), and levels of immunity that are low below 20 years (5–9 years: approx. 0%; 10–19 years: 37%) and high above 20 years (20–44 years: 70% ; 45–64 years: 78%) [8]. The estimated attack rates and levels of immunity in our analyses are qualitatively—and to a reasonable extent also quantitatively—in agreement with attack rates and immunity rates based on serological data alone. The main quantitative difference is that estimates are more extreme when using serological data only, i.e. yielding higher attack rates in children and higher immunity estimates in adults and elderly. In our analyses, estimates are informed mainly by serological data, but also constrained by the hospitalization data and by contact patterns.

Our estimates and earlier estimates of the attack rate of the 2009 pandemic in The Netherlands are highly skewed, and range from up to 60% in young children (5–9 years) down to 0–1% in elderly [7]. The 2009 pandemic virus is structurally very similar to the 1918 H1N1 virus [23], and it has been shown that people exposed to pre-1957 H1N1 viruses have a substantial degree of pre-existing immunity to A/2009 H1N1-like viruses [24,25]. Our finding of high levels of immunity combined with relatively high probability of hospitalization after infection in older adults (approaching 1% in our main analyses) indicates that pre-existing immunity played a crucial role keeping the overall public health impact of the 2009 pandemic low [7].

## Supplementary Material

Supplementary Data

## Supplementary Material

Model pseudocode

## Supplementary Material

Supplementary Tables
