# Supplementary material for: Joint modelling of serological and hospitalization data reveals that high levels of pre-existing immunity and school holidays shaped the influenza A pandemic of 2009 in The Netherlands
Source: J R Soc Interface. 2015 Feb 6;12(103):20141244. doi: 10.1098/rsif.2014.1244 (PMC4305427; doi:10.1098/rsif.2014.1244)
Supplement: Model pseudocode [file rsif20141244supp2.doc]

**Overview of the Markov Chain Monte Carlo method**

Here we give a brief description of the steps taken to obtain a sample from the posterior density. Details on how to calculate the solution of the transmission model, the likelihood of the serological data, and the likelihood of the hospitalisation incidence data have been omitted for transparency. Here the focus is on how the individual elements are combined. Throughout, we use a simple random-walk Metropolis algorithm for updating.

A. Initialise the parameter vector *X^current^* and calculate the likelihood *L^current^* (using steps 3-6 below). This forms the starting point for the MCMC.

B. Repeat the steps below until convergence and a proper sample from the posterior distribution has been obtained:

1. Sample all parameters or (for better mixing) a subset of parameters from normal distributions centred around their current value: *X^candidate^~N(X^current^,sd)*. The standard deviations are tuned to achieve optimal mixing;
2. Check if the sampled parameter values are within admissible intervals. If not, resample.
3. Use the parameters of the SEIR transmission model to calculate incidence curves $i_{a}\left(t\right)$ and final sizes $R_{a}(\infty)-R_{a}(0)$ of the epidemic in each age group *a*;
4. Use the modelled incidence curves $i_{a}\left(t\right)$ and the hospitalisation rates $\psi_{a}$ to calculate the log-likelihood of the hospitalisation data. Throughout the main text we assume that hospitalisation data are Poisson distributed with means determined by the modelled incidence;
5. Use the parameters that determine the mixture distributions $\left(\mathbf{\theta}_{\mathbf{sus}},\mathbf{\theta}_{\mathbf{imm}},\mathbf{\theta}_{\mathbf{inf}}\right)$ together with the vectors $\mathbf{p}$ and $\mathbf{q}$ containing the age-specific weights of the distributions to calculate the log-likelihood of the serological data. In the calculations, the weights of the immune component are set by the immunity levels in the transmission model (i.e. $p_{a}=R_{a}(0)$), and the weights of the infected component are set by the final size of the epidemic
   (i.e. $q_{a}=R_{a}(\infty)-R_{a}(0)$). Together, steps 4 and 5 link the serological and hospitalisation data through the transmission model;
6. Add the log likelihoods of the hospitalisation and serological data to obtain an updated log‑likelihood *L^candidate^*;
7. Evaluate the likelihood using the proposed parameters against the current likelihood and accept the candidate sample if *L^candidate^ / L^current^ < U* where *U~Uniform(0,1)*.
