# Supplementary material for: Joint modelling of serological and hospitalization data reveals that high levels of pre-existing immunity and school holidays shaped the influenza A pandemic of 2009 in The Netherlands
Source: J R Soc Interface. 2015 Feb 6;12(103):20141244. doi: 10.1098/rsif.2014.1244 (PMC4305427; doi:10.1098/rsif.2014.1244)
Supplement: Supplementary Tables [file rsif20141244supp3.doc]

**SUPPLEMENTARY TABLES**

**Joint modelling of serological and hospitalisation data reveals that high levels of pre-existing immunity and school holidays shaped the influenza A pandemic of 2009 in the Netherlands**

Dennis te Beest, Paul J Birrell, Jacco Wallinga, Daniela DeAngelis, Michiel van Boven

Supplementary Table 1. The contact matrix as used in the transmission model. Shown are the average number of contacts per day that persons in a certain age group have with persons of other age groups. Data are from the POLYMOD study (Mossong et al. 2008; see main text).

|  | Age Group (years) | | | | |
| --- | --- | --- | --- | --- | --- |
|  | 1-4 | 5-9 | 10-19 | 20-64 | 65+ |
| 1-4 | 3.2 | 1.5 | 0.3 | 5.2 | 0.3 |
| 5-9 | 1.5 | 8.4 | 1.7 | 5.8 | 0.4 |
| 10-19 | 0.2 | 0.9 | 10.0 | 5.7 | 0.4 |
| 20-64 | 0.5 | 0.6 | 1.1 | 10.1 | 0.7 |
| 65+ | 0.2 | 0.2 | 0.5 | 4.0 | 2.2 |

Supplementary Table 2. Estimates of the fraction of the population infected at *t=*0, and of the mixing distributions of the model that does not include the school holiday (cf. Table 1).

| Parameter | | Estimate | (95%CrI) |
| --- | --- | --- | --- |
| Fraction infected at *t=0* |  | 0.000044 | (0.000033-0.000059) |
| Susceptible distribution | Mean | 1.04 | (0.05-2.51) |
|  | SD | 0.54 | (0.02-1.25) |
| Immune distribution | Mean | 3.75 | (3.55-3.93) |
|  | SD | 0.92 | (0.79-1.08) |
| Infected distribution | Mean | 5.28 | (4.67-5.75) |
|  | SD | 0.84 | (0.64-1.39) |

Supplementary Table 3. Estimates of the fraction of the population infected at *t=*0, and of the mixing distributions of the model that includes the school holiday (cf. Table 2).

| Parameter | | Estimate | (95%CrI) |
| --- | --- | --- | --- |
| Fraction infected at *t=0* |  | 0.000014 | (0.000008-0.000026) |
| Susceptible distribution | Mean | 1.04 | (0.05-2.53) |
|  | SD | 0.48 | (0.02-1.24) |
| Immune distribution | Mean | 3.70 | (3.49-3.89) |
|  | SD | 0.95 | (0.81-1.1) |
| Infected distribution | Mean | 5.27 | (4.63-5.72) |
|  | SD | 0.85 | (0.66-1.41) |

Supplementary Table 4. Parameter estimates of the model that includes the holiday effect, and in which the immune mixture distribution is forced to have at least 95% of its density above the detection limit (cf. Table 2).

| Parameter | Age group (years) | Estimate | (95% CrI) |
| --- | --- | --- | --- |
| Fraction immune before | 1-4 | 0^1^ |  |
| the pandemic | 5-9 | 0.02 | (0-0.08) |
|  | 10-19 | 0.22 | (0.14-0.30) |
|  | 20-64 | 0.52 | (0.43-0.61) |
|  | 65+ | 0.84 | (0.64-0.91) |
| Infection attack rate | 1-4 | 0.30 | (0.26-0.35) |
|  | 5-9 | 0.57 | (0.52-0.61) |
|  | 10-19 | 0.39 | (0.33-0.45) |
|  | 20-64 | 0.12 | (0.09-0.18) |
|  | 65+ | 0.02 | (0.01-0.05) |
| Reduction of transmission | 5-9 | 0.54 | (0.25-0.86) |
| during school holiday | 10-19 | 0.13 | (0.01-0.37) |
| Basic reproduction number |  | 1.9 | (1.8-2.1) |
| Reproduction number at the start of the pandemic |  | 1.40 | (1.35-1.47) |
| Probability of hospitalisation | 1-4 | 0.00090 | (0.00074-0.0011) |
|  | 5-19 | 0.00031 | (0.00027-0.00036) |
|  | 20-64 | 0.00064 | (0.00045-0.00093) |
|  | 65+ | 0.0036 | (0.0014-0.0072) |
| Fraction infected at *t=0* |  | 2.0E-05 | (1.1E-5 – 3.4E-5) |
| Susceptible distribution | Mean | 2.40 | (1.73 – 2.73) |
|  | SD | 0.47 | (0.21 – 0.84) |
| Immune distribution | Mean | 4.13 | (4.03 – 4.25) |
|  | SD | 0.67 | (0.61 – 0.74) |
| Infected distribution | Mean | 5.19 | (4.73 – 5.61) |
|  | SD | 0.80 | (0.55 – 1.17) |

Parameter estimates are represented by the medians of the posterior distribution. ^1^Pre-pandemic immunity is assumed to be absent in the age group 1-4 years.

Supplementary Table 5. Parameter estimates of the model that includes the holiday effect, and in which the maximal differences between age groups of hospitalisation probabilities are doubled in comparison with the results presented in Table 2.

| Parameter | Age group (years) | Estimate | (95% CrI) |
| --- | --- | --- | --- |
| Fraction immune before | 1-4 | 0^1^ |  |
| the pandemic | 5-9 | 0.06 | (0.00-0.21) |
|  | 10-19 | 0.27 | (0.18-0.38) |
|  | 20-64 | 0.78 | (0.63-0.86) |
|  | 65+ | 0.95 | (0.78-0.98) |
| Infection attack rate | 1-4 | 0.27 | (0.22-0.31) |
|  | 5-9 | 0.53 | (0.44-0.58) |
|  | 10-19 | 0.34 | (0.28-0.40) |
|  | 20-64 | 0.04 | (0.02-0.08) |
|  | 65+ | 0.004 | (0.001-0.02) |
| Reduction of transmission | 5-9 | 0.50 | (0.27-0.76) |
| during school holiday | 10-19 | 0.08 | (0.00-0.26) |
| Basic reproduction number |  | 2.2 | (2.0-2.6) |
| Reproduction number at the start of the pandemic |  | 1.41 | (1.36-1.48) |
| Probability of hospitalisation | 1-4 | 0.0010 | (0.00083-0.0013) |
|  | 5-19 | 0.00035 | (0.00030-0.00043) |
|  | 20-64 | 0.0021 | (0.00097-0.0037) |
|  | 65+ | 0.017 | (0.0037-0.053) |
| Fraction infected at t=0 |  | 1.5E-05 | (8.3E-6 – 2.7E-5) |
| Susceptible distribution | Mean | 1.05 | (0.05-2.55) |
|  | SD | 0.48 | (0.02-1.29) |
| Immune distribution | Mean | 3.62 | (3.41-3.85) |
|  | SD | 0.99 | (0.83-1.16) |
| Infected distribution | Mean | 5.27 | (4.58-5.73) |
|  | SD | 0.88 | (0.68-1.47) |

Parameter estimates are represented by the medians of the posterior distribution. ^1^Pre-pandemic immunity is assumed to be absent in the age group 1-4 years.

Supplementary Table 6. Parameter estimates of the model that uses a micro array titre threshold of 20, and includes the effect of the school holiday. In this model every person with a titre higher than 20 is assumed to be immune in the pre-pandemic survey, and immune or infected in the post-pandemic survey. We use a binomial likelihood to calculate the probability that a certain number of individuals (given by the increase in persons above the threshold) had been infected, given the attack rate from the epidemic model (Birrell et al. 2011; cf. Table2).

| Parameter | Age group (years) | Estimate | (95% CrI) |
| --- | --- | --- | --- |
| Fraction immune before | 1-4 | 0^1^ |  |
| the pandemic | 5-9 | 0.08 | (0-0.22) |
|  | 10-19 | 0.30 | (0.20-0.40) |
|  | 20-64 | 0.62 | (0.51-0.69) |
|  | 65+ | 0.86 | (0.73-0.92) |
| Infection attack rate | 1-4 | 0.32 | (0.28-0.38) |
|  | 5-9 | 0.55 | (0.46-0.61) |
|  | 10-19 | 0.36 | (0.29-0.42) |
|  | 20-64 | 0.10 | (0.06-0.15) |
|  | 65+ | 0.02 | (0.01-0.04) |
| Reduction of transmission | 5-9 | 0.65 | (0.39-0.94) |
| during school holiday | 10-19 | 0.16 | (0.01-0.42) |
| Basic reproduction number |  | 2.2 | (2.0-2.5) |
| Reproduction number at the start of the pandemic |  | 1.44 | (1.39-1.50) |
| Probability of hospitalisation | 1-4 | 0.00084 | (0.00069-0.0010) |
|  | 5-19 | 0.00034 | (0.00028-0.00041) |
|  | 20-64 | 0.00080 | (0.00053-0.0013) |
|  | 65+ | 0.0040 | (0.0019-0.0085) |

Parameter estimates are represented by the medians of the posterior distribution. ^1^Pre-pandemic immunity is assumed to be absent in the age group 1-4 years. See Table 2 for comparison with mixture model analysis.

Supplementary Table 7. Parameter estimates of the model that uses a micro array titre threshold of 40, and includes the effect of the school holiday. In this model every person with a titre higher than 40 is assumed to be immune in the pre-pandemic survey, and immune or infected in the post-pandemic survey. We use a binomial likelihood to calculate the probability that a certain number of individuals (given by the increase in persons above the threshold) had been infected, given the attack rate from the epidemic model (Birrell et al. 2011; cf. Table 2).

| Parameter | Age group (years) | Estimate | (95% CrI) |
| --- | --- | --- | --- |
| Fraction immune before | 1-4 | 0^1^ |  |
| the pandemic | 5-9 | 0.05 | (0-0.19) |
|  | 10-19 | 0.28 | (0.18-0.38) |
|  | 20-64 | 0.36 | (0.28-0.46) |
|  | 65+ | 0.72 | (0.56-0.85) |
| Infection attack rate | 1-4 | 0.36 | (0.33-0.39) |
|  | 5-9 | 0.58 | (0.48-0.62) |
|  | 10-19 | 0.38 | (0.31-0.44) |
|  | 20-64 | 0.22 | (0.17-0.27) |
|  | 65+ | 0.05 | (0.02-0.08) |
| Reduction of transmission | 5-9 | 0.69 | (0.37-0.97) |
| during school holiday | 10-19 | 0.22 | (0.02-0.56) |
| Basic reproduction number |  | 1.9 | (1.7-2.1) |
| Reproduction number at the start of the pandemic |  | 1.40 | (1.36-1.45) |
| Probability of hospitalisation | 1-4 | 0.00076 | (0.00066-0.00089) |
|  | 5-19 | 0.00032 | (0.00026-0.00039) |
|  | 20-64 | 0.00036 | (0.00029-0.00048) |
|  | 65+ | 0.0014 | (0.00084-0.0029) |

Parameter estimates are represented by the medians of the posterior distribution. ^1^Pre-pandemic immunity is assumed to be absent in the age group 1-4 years. See Table 2 for comparison with mixture model analysis, and Supplementary Table 6 for comparison with analysis that uses a threshold of 20.
